# Fibrous Tumour

**Published:** 1854-12

**Authors:** 


Fibrous Tumour.—A patient presented a large fibrous tumour of the neck, arising from the transverse processes of the vertebræ. It was of such a size, and had such relations, that ablation was thought impossible. M. Maisonneuve removed it by reflecting the skin, and thereby dividing the tumour into two parts, and dissecting each separately. By this plan this surgeon has removed large fibrous tumours of the uterus, which could not have been taken out in entire masses.—*Bull. Gén. de Thér.*

